# Embedding an engagement exercise into an administrative data research project: An example focussing on Gypsy, Traveller and Roma young people and their families

**DOI:** 10.23889/ijpds.v8i2.2337

**Published:** 2023-09-14

**Authors:** Polly Vizard, Polina Obolenskaya

**Affiliations:** 1 Centre for Analysis of Social Exclusion, London School of Economics, London, United Kingdom; 2 City University, London, United Kingdom

## Objectives

The presentation reports findings from the ADR funded project ‘Testing and demonstrating the value of the Growing Up in England dataset: Gypsy, Traveller and Roma case study’. It will (1) address the rationale for embedding an engagement exercise in this project; (2) set out methodology and findings; and (3) draw broader implications for administrative data research.

## Methods

Two engagement workshops were co-organised with the civil society organisation Friends, Families and Travellers (FFT). Recruitment was via FFT outreach networks and young people aged 14-29 and their broader family groups were invited to attend. Participants included Romany Gypsies, Traveller and Roma young people and family groups, ensuring diversity of experiences and perspectives. Informed consent was obtained from all participants (and from parents/carers for under 18s). The purpose of the engagement exercise was to hear directly from Gypsy, Traveller and Roma young people about issues raised by our analysis of the newly linked Growing Up in England (GUiE) dataset.

## Results

Feedback from the workshops explores the educational experiences of Gypsy, Traveller and Roma young people and their families and provides important insights into barriers to educational progression and unmet needs for support. Additionally, feedback from the workshops provides new insights into the views, perspectives and experiences of Gypsy, Traveller and Roma young people and their families relating to administrative data collection and data use. One key concern highlighted by workshop participants is that the disclosure of Gypsy, Traveller and Roma identity in administrative data collected by schools, hospitals and other public services could result in discrimination or other forms of adverse or hostile treatment.

## Conclusion

The findings from the engagement exercise have important implications for our GUiE project and for broader reflection on public attitudes to administrative data. Additionally, the exercise enabled us to address NSDEC recommendations on engagement. However, engaging with Gypsy, Traveller and Roma young people has involved specialist input, time, funding and additional ethics review, which all require careful consideration and planning.

